# Analysis of Total Phenols, Sugars, and Mineral Elements in Colored Tubers of *Solanum tuberosum* L.

**DOI:** 10.3390/foods9121862

**Published:** 2020-12-14

**Authors:** Piret Saar-Reismaa, Katrin Kotkas, Viive Rosenberg, Maria Kulp, Maria Kuhtinskaja, Merike Vaher

**Affiliations:** 1 School of Science, Tallinn University of Technology, Akadeemia tee 15, 12618 Tallinn, Estonia; piret.saar1@taltech.ee (P.S.-R.); maria.kulp@taltech.ee (M.K.); maria.kuhtinskaja@taltech.ee (M.K.); 2 Estonian Crop Research Institute, J. Aamissepa 1, 48309 Jõgeva, Estonia; katrin.kotkas@etki.ee (K.K.); viiverosenberg@gmail.com (V.R.)

**Keywords:** colored potato tubers, total phenols, anthocyanins, antioxidants, saccharides, nutrition, microelements

## Abstract

The use of colored tubers of *Solanum tuberosum* L. is growing worldwide due to their health benefits and attractive color. The positive health effects of purple-fleshed tubers are a result of anthocyanins and various phenolic compounds. The aim of this study was to evaluate and compare variety Blue Congo and its cross-breeds of Desiree and Granola to yellow-fleshed tubers. The concentration of total phenols, anthocyanins, sugars, and mineral elements were evaluated in all tubers. The results showed differences between all tested materials, with largest differences in sugar content. Moreover, the results confirmed the preservation of health improving compounds of Blue Congo when cross-bred with yellow-fleshed tubers. The total phenolic content and anthocyanin concentrations of all analyzed tubers were above the comparison yellow ones.

## 1. Introduction

Worldwide, potatoes (*Solanum tuberosum* L.) are the fourth important food crop after wheat, rice, and maize. Potatoes are grown in cool-temperature regions, in mountainous areas as well as at higher altitudes in the tropic. Potato tubers are a rich source of high-value protein, carbohydrates, essential vitamins, minerals, and trace elements. The average range of a potato tuber composition is as follows: starch (10–18%) having 22–30% amylose content, total sugars (1–7%), protein (1–2%), fiber (0.5%), lipids (0.1–0.5%), vitamin A (trace/100 g of fresh weight (FW)), vitamin C (30 mg/100 g of FW), various trace minerals, and glycoalkaloids (1–3 mg/100 g of FW) [1]. 

Potato tubers are known to be naturally high in potassium, up to 400 mg per 100 g fresh tubers [2], or 1.7% of dry matter [3]. The potassium from potatoes can help lower blood pressure due to its vasodilation effect [4]. The tubers also contain other essential microelements like iron and calcium, that are responsible for bone structure and strength, in addition, zinc, manganese, copper, and magnesium are represented, that regulate cell renewal, energy production, and are responsible for over-all immunity [5]. Consumption of potato tubers with high nutritional content contributes to fulfilling of the daily recommended intake of several essential elements. 

Over the past decade or so, colored-flesh potatoes have become more widely available to home gardeners, including potato tubers of blue, red, yellow, and white flesh. Purple-fleshed potatoes give the possibility to add color to the menu and additional nutrients to human diet. The addition of colored tubers adds the benefits from healthy antioxidants. Colored-flesh potatoes get their color from various pigments, which are antioxidants. Purple and rose-flesh potatoes contain the anthocyanin pigments, while yellow-colored flesh varieties contain carotenoids [6]. Purple-fleshed potatoes like Blue Congo get their color from common anthocyanins malvidin, peonidin, delphinidin, cyanidin, and petunidin [7]. Epidemiological evidence indicates health benefits from anthocyanins include improved eyesight and circulatory system function, benefits for diabetics, and anti-inflammatory, antiviral, and antimicrobial activity [8,9,10].

Potato tubers are also a great source of carbohydrates, which occur mainly in starch form [11] and are also used for industrial starch production. The starch content is directly related to the sugar content of tubers. As most potatoes are consumed after processing at high temperatures, the asparagine and glycose or fructose from the tubers can lead to acrylamide formation. This is due to Maillard reaction—a reaction between amino acid and sugar [12]. Moreover, higher levels of reducing sugars (glucose and fructose) as well as non-reducing sucrose in potato tubers may result in unfavorable browning or even a bitter taste [13]. Therefore, it is important to evaluate the sugar content in raw potato tubers, to determine if new varieties would produce high levels of acrylamide in processing. Moreover, potato tubers additionally contain myo-inositol, which is a sugar-like carbohydrate produced by most plants. Myo-inositol is important for phosphate storage and normal cell-to-cell communication and its metabolism is associated to diabetes [14]. 

The aim of the study was to evaluate the content of total phenols (TPs), anthocyanins, mineral elements, and sugars in tubers from variety Blue Congo, its seedlings, and cross-breeds with Granola and Desiree using various selective methods to create the plant material. 

## 2. Materials and Methods 

### 2.1. Studied Plant Material

The plant material, created by different methods in vitro was grown in a test-field in Saku, Estonia (local latitude 57°25′). The soil type of the experimental area was Calcaric cambisols according to the World Reference Base classification (EAO 2014) where the agrochemical indicators were as follows: pH 6.3 (ISO 10,390 [15]); soil carbon content Corg 3.3% (Tyurin method [16]) and concentration of soluble P and K being 114 and 161 mg/kg (Mehlich III method [17]). In spring time, before the cultivation, in the field the complex fertilizer Cropcare 8-11-23 500 kg/ha was used.

Detailed description of tubers is given in Table 1. Initial mini-tubers of the variety Blue Congo were received from Sweden by the potato grower in 1991. Mini-tubers grown in green-house were eradicated on virus infection by using thermotherapy and meristem-plants were created in vitro [18]. 

Botanical seeds of the variety Blue Congo (1–7 in Table 1) were cultivated into the test-tubes in sterile condition, regenerated plants were multiplied in vitro and grown in the test-field. In 2013, the plants of the variety Blue Congo were pollinated in field conditions with the variety Desiree (8, 9) and with the variety Granola (10–15). The tubers of the variety Desiree are red skinned with yellow flesh, and the tubers of the variety Granola are yellow skinned with blanched yellow flesh.

New tissue cultures from the plants preserved in vitro were created on years 2004–2007. On the base of field results, three best meristem clones were selected (16–18).

As a comparing variety group, two commercial varieties were included. Variety Teele tubers are with bright yellow skin and yellow flesh and variety Laura tubers are with red skin with dark yellow flesh. Comparison with super-market purple-fleshed sweet potato tubers (*Ipomoea batatas* L.) were also included.

### 2.2. Extraction and Dry-Weight

From each genotype (plant material type) (Table 1) 5 tubers were selected. The tubers were washed with distilled water, dried at room temperature and homogenized (Nutribullet, Los Angeles, CA, USA). The homogenates were divided into aliquots and stored at 4 °C for further analysis. The aliquots were used for determination of dry weights (DW), microelements, and for making extracts for analysis of total phenols (TPs), anthocyanins, and naturally occurring sugars. 

For evaluation of TPs and anthocyanins the following extraction procedure was conducted: 5 g of tuber homogenate was mixed with 25 mL 80% (*v/v*) methanol in a 50 mL graduated tube. The mixture was allowed to stand with intermittent shaking for 1 h at room temperature in the dark. After that the mixture was subjected to ultrasonication (Sonorex digital 10P, Bandelin, Berlin, Germany) for 30 min at 30 °C. After which it was centrifugated (EBA 200S, Hettich, Westphalia, Germany) at 8000 rpm/min for 15 min and the supernatant was filtered through 0.45 µm Minisart^®^ Syringe Filter (Sartorius, Goettingen, Germany) and stored at 4 °C. The carbohydrate extraction was achieved in the ultrasonic bath with 50% (*v/v*) aqueous methanol from 5 g of homogenate using the same procedure as in case of extraction of polyphenols.

The dry weight of the tubers was measured by drying ~1 g of the homogenate at 105 °C until constant weight using an Ohaus Moisture Analyzer MB90 (Parsippany, NJ, USA) in triplicates. The content of dry matter ranged from 15.8 to 31.5% for examined potato tubers and was 34.9% for the purple sweet potato tuber.

### 2.3. Determination of Total Phenols and Anthocyanins

The concentration of the total phenolic compounds was determined for each extract by an adapted micro-scale protocol for the Folin–Ciocalteu colorimetric method [19,20]. In brief, phenolic groups are oxidized by phosphomolybdic and phosphotungstic acids in Folin–Ciocalteu reagent, forming a green–blue complex detectable at 765 nm. 50 μL of each tuber extract solution of an appropriate concentration was mixed with 1350 µL of water, 100 µL Folin–Ciocalteu reagent and 500 μL of Na_2_CO_3_ (20% *w/w*). The absorbance at 765 nm was measured after 2 h reaction at room temperature (in the dark) with a Cary 50 Bio UV–vis spectrophotometer (Palo Alto, Varian, CA, USA). The hydro-methanolic gallic acid solution was freshly prepared in a series of concentrations (0.3–3 mM) and tested in parallel to establish the calibration curve. The total phenolic content of each potato extract was calculated as milligrams of gallic acid equivalent per g dry sample (mg GAE/g of DW). 

The total content of non-hydrolyzed anthocyanins was measured using the pH differential method described by Albishi et al. [21] with minor modification. Monomeric anthocyanin pigments reversibly change color with change in pH. The colored oxonium form exist at pH 1.0, and the colorless hemiketal form predominates at pH 4.5. The difference in the absorbance at 520 nm is proportional to the pigment concentration. 400 μL of potato tuber or 200 μL of sweet potato tuber extract and 600 or 800 μL of 25 mM potassium chloride buffer (pH 1.0) and 400 mM sodium acetate buffer (pH 4.5) respectively were mixed. The mixtures were left at room temperature for 1 h (in the dark). The absorbance was measured at 520 nm and 700 nm against a blank cell filled with 80% MeOH in buffer. The results were expressed as mg of cyanidin-3-glucoside equivalents per kg dry sample (mg CGE/kg of DW). 

The experiments were carried out in triplicates, and the results are reported as the mean ± standard deviation.

### 2.4. Capillary Electrophoretic Analysis of Natural Sugars

Capillary electrophoresis (CE) was performed using an Agilent 3D CE instrument (Agilent Technologies, Santa Clara, CA, USA) equipped with a diode array UV/Vis detector. Uncoated fused silica capillary with effective length of 71.5 cm and i.d. of 50 μm was employed. The optimized conditions for the analysis: temperature of the capillary was 16 °C, applied voltage was +17 kV and samples were injected under 35 mbar pressure for 10 s. 130 mM NaOH containing 36 mM Na_2_HPO_4_ (pH 12.6) was used as a background electrolyte (BGE). The wavelength for detection was 270 nm [22]. Identification of the sugars was done by standard addition method, the standard solutions of sucrose, D-(+)-maltose, D-(+)-glucose, D-(−)-fructose, sugar alcohol myo-inositol, and sodium hydroxide were from Sigma (Darmstadt, Germany). Milli-Q water (Millipore S. A, Molsheim, France) was used for all solutions of standards, background electrolyte (BGE), and dilution of samples.

### 2.5. Atomic Absobrance Analysis of Microelements

The stock atomic spectroscopy standard solutions (1000 mg/L) Cu, Zn, Mn, Fe, Mg, Ca, Se, and K were purchased from Fluka, Buchs, Switzerland. Spectra AA 220F and 220Z atomic absorption spectrometers (Varian, Mulgrave, Australia) equipped with a side-heated GTA-110Z graphite atomizer, a Zeeman effect background correction, and an integrated autosampler were used. Graphite tubes with coating and platforms made of pyrolytic graphite were used throughout the work. Argon of 99.99% purity (AGA, Helsinki, Finland) was used as the purge gas. Acetylene of 99.99% purity (AGA, Helsinki, Finland) was used as the fuel gas in flame atomic absorption spectroscopy. For the determination of total mineral element constituents ~1 g of potato tubers homogenate was mineralized with 4 mL of concentrated nitric acid and 1 mL of concentrated hydrogen peroxide in 50 mL plastic tubes at temperature 80 °C for 5 h. After cooling down, the solution was transferred to volumetric flasks (15 mL) with ultrapure water. All the experiments were made in triplicates. The concentrated nitric acid and hydrogen peroxide were from Sigma (Darmstadt, Germany).

### 2.6. Statistical Analysis

The statistical analysis was conducted using Microsoft Excel and R version 4.0.2. The significant variations were evaluated using the Excel built-in data analysis package (*t*-test two samples, *p* = 0.05) and the principal component analysis (PCA) was carried out and visualized in R 4.0.2 x64.

## 3. Results and Discussion

### 3.1. Total Phenlos and Anthocyanins

**Figure 1:**
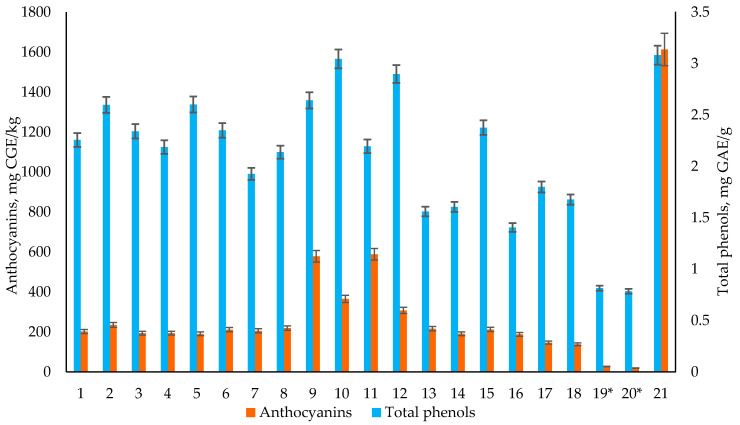
The concentrations of anthocyanins and total phenols in samples 1–21. All concentrations are given for dry weight. * *p* < 0.05 for total phenols and anthocyanin concentration between yellow-fleshed and colored potato tubers.

To evaluate the possible positive health influences of colored tubers, the total phenols (TPs) and anthocyanin concentrations were evaluated compared to sweet potato and two yellow-fleshed tubers. The total phenols were evaluated in all tubers and compared as concentration of gallic acid equivalents mg/g of dry weight (mg GAE/g of DW). All sample tubers (Sample 1–20) as well as the sweet potato sample (Sample 21) showed TP concentrations between 0.8 and 3.1 mg GAE/g of DW as shown in Figure 1. 

The biggest difference occurred between all colored tubers (Samples 1–18) and the yellow varieties (Samples 19–20), where the yellow-fleshed tubers had significantly (*p* < 0.05) lower TP concentrations. The values for all the colored potato tubers were from 1.4 to 3.1 mg GAE/g of DW, but for the yellow fleshed tubers only 0.8 mg GAE/g of DW, showing an increase of 75–175% of TPs for purple-fleshed varieties. These results are in accordance with previous studies of colored tubers, that show an increase of various phenolic acids, coumarins, and flavonoids in purple fleshed potatoes compared to yellow or white tubers [6,23]. The results also show that crossbreeding with potatoes with yellow flesh do not significantly decrease the variation of TPs in tubers, allowing for more versatile and specific breeding. The TP concentration in the purple sweet potato tuber did not have a remarkable difference compared to all other purple-fleshed tubers. 

Another important evaluation criterion for the health-benefit of the purple-flesh potato is the content of anthocyanins. The same samples were analyzed for anthocyanins with results shown in Figure 1. As anthocyanins are directly linked to the coloration of the flesh, the concentrations of anthocyanins in yellow-fleshed sample tubers, samples 19–20, were the smallest, showing a statistical difference to all other tubers (*p* < 0.05). All colored potato tubers had anthocyanin concentrations from 138.6 to 588.5 mg CGE/kg of DW, compared to yellow fleshed tubers concentrations around 20 mg CGE/kg of DW. Such results are to be expected as Jansen and Flamme [24] have previously shown, that whole violet and violet/white fleshed tubers had anthocyanin concentrations from 181 to 1570 mg/kg of FW, while white and yellow fleshed tubers only had 4–82 mg/kg of FW of anthocyanins. The purple sweet potato had over six times higher concentration of 1613.4 mg CGE/kg of DW compared the average of 254.9 mg CGE/kg of DW from Blue Congo and its examined accessions varieties. Unlike TPs, the anthocyanins had a small increase in the average concentration in Blue Congo cross-breeds with Granola, implying the positive effect from the breed Granola. 

### 3.2. Analysis of Sugars

The sugar content of potatoes is an important quality indicator. It has been shown that the tuber sugar content can vary by genotype, but is largely influenced by the storage and treatment, even more so at temperatures below 10 °C [13]. All analyzed samples were therefor stored at similar temperature to avoid any environment factor as a variable. 

**Figure 2:**
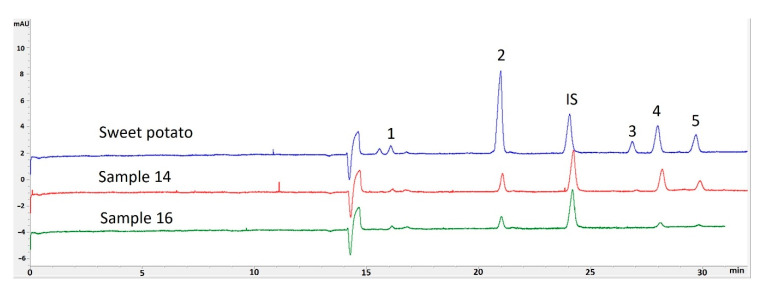
Electropherograms of sugar analysis from sweet potato tuber Sample 21 (blue), purple-fleshed cross-breed with Granola Sample 14 (red) and a meristem clone of Blue Congo sample 16 (green). Identification 1—myo-inositol, 2—sucrose, IS—internal standard, 3—maltose, 4—glucose, 5—fructose.

The analysis was carried out using a CE with UV detection and lactose was used as an internal standard (IS). All samples were analyzed for myo-inositol, sucrose, maltose, glucose, and fructose. Examples of analyzed tuber electropherograms are shown in Figure 2. 

**Figure 3:**
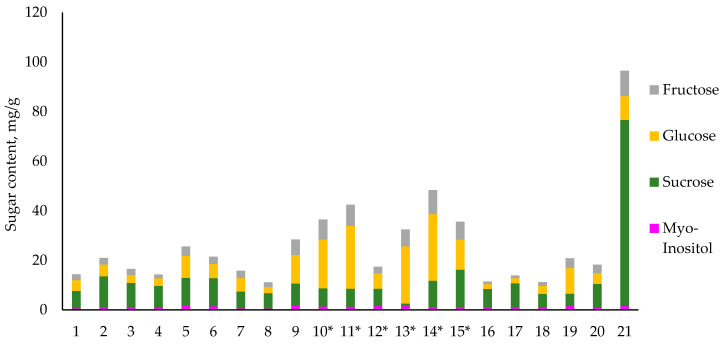
The total content of sugars from analyzed potato tubers Samples 1–18 correspond to purple-fleshed tubers, samples 19–20 to yellow-fleshed tubers and sample 21 to a purple sweet potato tuber. * *p* < 0.05 for total sugars between cross-breeds of Blue Congo with Granola compared to all other potato tubers.

None of the potato tuber samples contained maltose above the detection limit. The only sample to contain maltose was the purple sweet potato tuber, which was to be expected as the sweet taste of the tuber is due to maltose concentration [25]. To better evaluate the overall sugar content of all analyzed tubers, the fructose, glucose, sucrose, and myo-inositol concentrations were summed in a bar chart shown in Figure 3. 

The overall sugar content in all potato tubers ranged from 10.3 mg/g to 47.1 mg/g with an average of 21.6 mg/g. These results are in accordance with other published reports of total sugar concentrations in potato tubers that ranged from 7.5 to 74.1 mg/g of DW [26], and are also in accordance to climatically similarly grown potato tubers, where sucrose, glucose, and fructose ranged from 6.4 to 21.8, 2.3 to 29.7, and 1.2 to 25.4 mg/g of DW, respectively [27]. The sweet potato tuber total sugar concentration was 95.0 mg/g, which was mainly due to high concentration of sucrose. The lowest sugar concentrations were observed in the Blue Congo meristem clones, that averaged only 11.0 mg/g of total sugars. 

Additionally, there was a significant difference (*p* < 0.05) in the samples 9–15 corresponding mainly to the cross-breeds of Blue Congo and Granola with an average sugar content of 34.1 mg/g, while all other potato tubers had an average of only 16.3 mg/g. As the sugar content is used as a quality factor, the higher sugar concentration may result in unacceptably brown and bitter food products. Thus, the higher amounts found in Granola cross-breeds make such cultures less-favorable compared to the original Blue Congo variety for fried products. The cross-breeds with Desiree did not show such tendencies. 

### 3.3. Microelements in Tubers

The dispersion of various elements throughout the potato tubers is mixed, as some elements have higher concentration in the skins, while others in the flesh; moreover, there is some research that shows heterogeneous distribution between the stem and distal end of the tubers [28]. To better evaluate overall concentrations, whole tubers were washed and grounded for the analysis. Selected microelements essential for living organisms were evaluated using atomic absorbance spectroscopy (AAS). In total, eight elements were measured from all samples including copper, zinc, manganese, iron, magnesium, calcium, potassium, and selenium. All levels of Se were below the detection limit of the method (<20 mg/kg of DW). The results for all other elements are presented in Table 2 below. 

Potato tubers are best-known for their high potassium content as it is an essential element for the acid–base regulation as well as heart, liver, nerve, and muscle functioning [29]. The potassium concentration was the highest, ranging from 10.35 to 22.83 g/kg of DW, with an average of 16.4 g/kg of DW. These results are comparable to non-organic K levels determined in potato tubers [30].

The zinc concentration varied from 9.8 to 26.0 mg/kg of DW, matching with previous results of fertilized and organic Zn concentrations [5,31,32]. The concentrations did not vary depending on the flesh coloration of the tuber, but the purple sweet potato tuber had a significantly lower Zn concentration of only 8.2 mg/kg of DW. 

The Fe concentrations of analyzed tubers varied from 48.3 to 133 mg/g of DW, which is similar to works by Andre et al. [28]. The Fe levels in samples 19 and 20 as well as in the sweet potato were the lowest compared to colored-flesh tubers. The Fe found in potato tubers is considered to be non-heme iron and therefor considered a valuable source of iron for the human diet. The lack of iron can cause severe health problems, including impaired development in adolescence and reduced work capacity, making potatoes useful sources for such nutrients. Moreover, as potatoes also contain vitamin C, which increases the iron uptake from potatoes, the health benefits are significant against anemia. 

Calcium is needed for skeletal and neural functioning, as well as metabolism. Although, the Ca content is insufficient for marginal dietary benefits, the tuber quality and storage capacity is evaluated on the basis of it [33]. The Ca levels averaged at 470 mg/kg of DW, being similar to previously reported values, with the highest concentration of 729 mg/kg of DW and lowest of 270 mg/kg of DW. There were no significant variances between different breeding varieties or colored and yellow-fleshed tubers, nor sweet potato tuber. 

Mn concentrations were relatively low, similar to organic cultivars, with the levels being from 5.3 to 12.0 mg/kg of DW. In general, higher magnesium levels were detected in the tubers of cross-breeds of Blue Congo and Granola. The overall levels varied from 629 to 1264 mg/kg of DW with an average of 1015 mg/kg of DW, which is comparable to both conventional and organically cultivated potato tubers, that averaged from 1183 to 1646 mg/kg of DW [5,32]. 

No obvious differences were seen between breeding varieties and cross-breeds of Blue Congo tubers. The differences were observed with a purple sweet potato tuber as well as yellow-fleshed tubers compared to purple-fleshed tubers. Most of the results correlate to each other as the main source of mineral concentrations is due to the soil and fertilization processes used, which were similar for all the tubers analyzed, except for the purple sweet potato tuber and store-bought varieties. 

### 3.4. Principal Component Analysis

**Figure 4:**
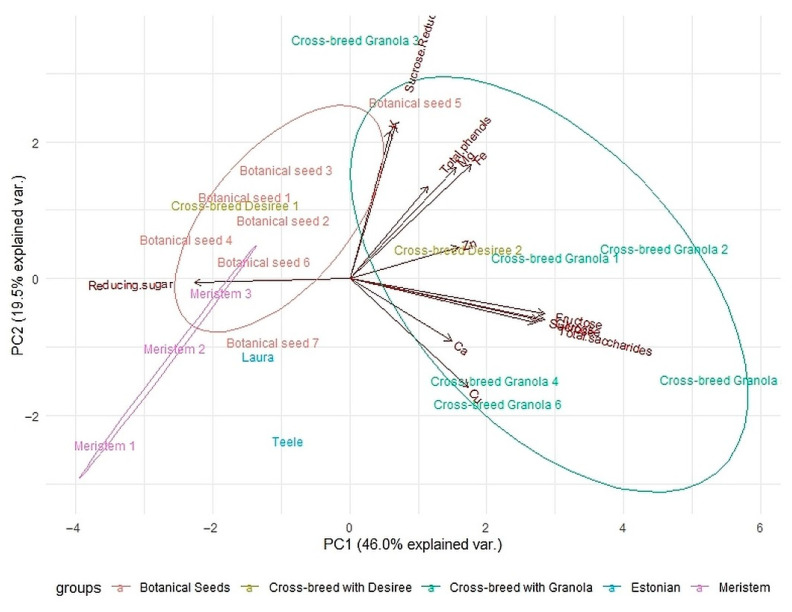
Principal component analysis (PCA) results of all analyzed 20 potato varieties and their mineral composition, natural sugars, total phenols, and anthocyanin content. Pink—meristem clones of Blue Congo, green—Blue Congo cross-breeds with Granola, blue—yellow-fleshed tubers, orange—Blue Congo botanical seeds, yellow—Blue Congo cross-breeds with Desiree.

A principal component analysis (PCA) was done to better evaluate the correlations between different breeding varieties and concentrations of mineral elements, natural sugars, and total phenols as well as anthocyanins. The sweet potato tuber results were excluded from the PCA analysis sweet potato is from another genus and it would be an outlier due to too different profile. The results of principal components 1 and 2 (PC1 and PC2) showed obvious groupings of all varieties as is seen in Figure 4. As the PC1 and PC2 had a total explained variance of 65.5%, the PCA was determined to be successful.

There were five main groups of potato tubers: botanical seeds of Blue Congo, meristem clones of Blue Congo, Blue Congo cross-breeds with Granola, Blue Congo cross-breeds with Desiree, and commercial varieties (Laura, Teele). The main distinguishable groupings were of botanical seeds of Blue Congo, which showed similarities to the meristem clones, but had almost no overlapping with cross-breeds with Granola. The main differences in the meristem clones and cross-breeds can be attributed to the reducing sugars, which make the Granola cross-breeds sugar rich as previously determined. Unfortunately, the high sugar content has links with higher copper and calcium concentrations, meaning that the possible benefits of Ca and Cu will not be obtained if the variety is deemed unsuitable as the sugar content may lead to cancerogenic acrylamide. To overcome this, there are studies that demonstrate the possibility to reduce the concentration of acrylamide formation even up to 93% by pretreatment of tubers [34]. The multi-step pretreatment may unfortunately diminish other nutrients. Moreover, the anthocyanin concentration is also in correlation with total saccharides, proving the value of such variety. 

Additionally, there was a correlation between higher concentration of Fe and total phenol concentration, which is in accordance with Brown’s previous results [31] where colored-flesh tubers have higher concentrations of TPs and Fe. The correlation of Fe and TPs also includes the higher concentration of Mg. The concentration of potassium was slightly correlated, showing cross-breeds with Desiree and some botanical seeds presenting with both positive characteristics. Interestingly, the botanical seeds of Blue Congo showed differences to the meristem clones of Blue Congo, with the botanical seeds having higher levels of nutrients. The yellow-fleshed tubers of Teele and Laura separated from all other tested material and had the smallest levels of anthocyanins, total phenols and additionally microelements, proving further that the purple-fleshed tubers have higher potential for a healthier alternative in the human diet.

## 4. Conclusions

The results of this work demonstrate differences of various potato tubers depending on their genotypes and varieties. Mainly Blue Congo tubers and their purple-fleshed cross-breeds were compared to yellow-fleshed potato tubers and purple sweet potato tubers. The levels of total phenolic compounds and anthocyanins found in purple-fleshed tubers were significantly higher compared to yellow-fleshed ones. Although, the Blue Congo and Granola cross-breed showed highest levels of sugars, the PCA analysis showed that additional beneficial anthocyanin concentration was in correlation with higher sugar content. In addition, the results confirmed that cross-breeding Blue Congo with a yellow-fleshed tuber does not diminish the positive benefits of high levels of phenolic compounds and anthocyanins. 

The results showed great potential to create versatile plant material with increased levels of specific TPs or anthocyanins, as well as adding to the knowledge of correlations of micronutrients to TPs, sugars, and anthocyanin concentrations. Thus, providing health benefits for various consumers to help with essential microelements as well as overall improvement of immune system and health. 

## Figures and Tables

**Table 1:** Descriptors for tubers of the variety Blue Congo seedlings and cross-breeds between Blue Congo with Desiree and Blue Congo with Granola.

Sample No	Material Number	Descriptors of Tubers		
		Skin Color	Flesh Color	Tuber’s Shape
**Botanical Seeds**				
1	25	Violet	Oak leaf form with white border around	Ovate
2	34	Violet	Violet with white border	Elongate, big
3	51	Dark violet	Dark violet marble	Ovate
4	53	Dark violet	Dark violet marble	Elongate
5	76	Pale violet	Pale reddish violet, white border	Ovate
6	89	Pale violet	White border, beautiful violet oak form	Round
7	116	Reddish violet	Pale reddish violet with white border	Ovate
**Cross-Breeding Blue Congo and Desiree**				
8	41	Dark violet	White border, violet vary-colored oak leaf form	Ovate
9	47	Dark pink	Pale pink border white in middle	Ovate
**Cross-Breeding Blue Congo and Granola**				
10	9	Dark violet	Dark violet pale border	Elongate big
11	16	Dark violet	Strong white border violet vary-color	Elongate big
12	28	Dark violet	Vary-color violet narrow border	Elongate-ovate
13	30	Dark pink	Netting pink violet	Uniform ovate
14	36	Dark pink	Netting blanched yellow middle	Ovate
15	41	Dark violet	Violet netting	Uniform ovate
**Meristem Clones of Blue Congo**				
16	40	Dark violet netting	Pale violet middle with pale border	Round
17	195	Dark violet netting	Dark violet with white border	Ovate
18	197	Dark violet netting	Pale violet with wider white border	Round
**Commercial Varieties**				
19	Teele	Yellow netting	Yellow	Round-ovate
20	Laura	Smooth dark red	Dark yellow	Round-ovate
21	Sweet Potato	Purple	Purple	Elongate

**Table 2:** Mineral composition of the tubers as determined by atomic absorbance spectroscopy (AAS) per dry weight (*n* = 3).

Sample No	Cu, mg/kg	Zn, mg/kg	Mn, mg/kg	Fe, mg/kg	Mg, mg/kg	Ca, mg/kg	K, g/kg
1	2.477	11.02	8.68	77.2	973.6	349.2	19.49
2	3.212	17.02	7.21	80.3	880.8	437.5	17.05
3	4.000	14.11	8.02	57.0	1253.6	554.1	22.83
4	2.146	12.14	7.18	61.2	921.4	583.0	17.36
5	3.016	17.31	8.63	109.9	1264.3	508.8	21.54
6	2.793	13.51	7.16	74.3	926.6	466.7	17.19
7	4.244	12.64	8.73	70.6	976.1	375.6	14.27
8	2.952	10.91	8.56	85.6	1082.8	623.9	17.78
9	4.116	26.01	9.25	86.1	865.3	331.8	11.81
10	4.335	15.69	12.04	85.0	1004.8	668.9	16.53
11	5.390	14.53	7.30	115.1	1138.4	591.8	18.86
12	1.994	14.64	9.89	133.0	1240.2	316.8	18.84
13	3.708	14.74	9.82	82.5	1087.7	377.8	14.19
14	5.937	18.35	9.37	120.3	1225.3	729.1	18.67
15	4.689	14.95	6.75	54.4	1029.6	392.2	13.71
16	3.943	9.81	10.29	56.2	692.4	319.4	10.35
17	4.688	14.20	6.74	58.9	921.0	501.3	14.47
18	3.153	13.32	7.86	77.7	1045.0	517.5	15.11
19	3.353	12.41	5.26	48.3	867.7	489.2	13.06
20	4.385	14.62	10.60	53.4	922.2	270.5	15.81
21	9.630	8.17	6.79	45.8	988.8	679.1	8.37
